# Improved Antimicrobial Properties of White Wastewater Protein Hydrolysate Through Electrodialysis with an Ultrafiltration Membrane (EDUF)

**DOI:** 10.3390/membranes15080238

**Published:** 2025-08-06

**Authors:** Diala Damen, Jacinthe Thibodeau, Sami Gaaloul, Steve Labrie, Safia Hamoudi, Laurent Bazinet

**Affiliations:** 1 Department of Food Sciences and Laboratoire de Transformation Alimentaire et Procédés ÉlectroMembranaires (LTAPEM, Laboratory of Food Processing and ElectroMembrane Processes), Université Laval, Quebec, QC G1V 0A6, Canada; diala.damen.1@ulaval.ca (D.D.); jacinthe.thibodeau.1@ulaval.ca (J.T.); 2 Institute of Nutrition and Functional Foods (INAF), Dairy Research Centre (STELA), Université Laval, Quebec, QC G1V 0A6, Canada; steve.labrie@fsaa.ulaval.ca; 3 Lactalis, Victoriaville, QC G6T 1S8, Canada; sami.gaaloul@ca.lactalis.com; 4 Department of Soil Sciences & Agri-Food Engineering, Centre in Green Chemistry & Catalysis, Centr’Eau, Université Laval, Quebec, QC G1V 0A6, Canada; safia.hamoudi@fsaa.ulaval.ca

**Keywords:** white wastewater, milk proteins, electrodialysis with filtration membrane, antimicrobial peptides, protein valorization

## Abstract

This study investigated white wastewater (WW) as a potential source of antimicrobial peptides, employing hydrolysis with Pronase E followed by separation through electrodialysis with ultrafiltration membranes (EDUF) to increase the value of dairy components within a circular economy framework. The WW hydrolysate was divided into two key fractions: the cationic recovery compartment (CRC) and the anionic recovery compartment (ARC). The EDUF process effectively separated peptides, with peptide migration rates reaching 6.83 ± 0.59 g/m^2^·h for CRC and 6.19 ± 0.66 g/m^2^·h for ARC. Furthermore, relative energy consumption (REC) increased from 1.15 Wh/g to 2.05 Wh/g over three hours, in line with trends observed in recent studies on electrodialysis energy use. Although 29 peptides were statistically selected from the CRC (20) and ARC (9) compartments, no antibacterial activity was exhibited against Clostridium tyrobutyricum and Pseudomonas aeruginosa; however, antifungal activity was observed in the feed and ARC compartments. Peptides from the ARC demonstrated activity against Mucor racemosus (MIC = 0.156 mg/mL) and showed selective antifungal effects against Penicillium commune (MIC = 0.156 mg/mL). This innovative approach paves the way for improving the recovery of anionic peptides through further optimization of the EDUF process. Future perspectives include synthesizing selected peptides and evaluating their antifungal efficacy against these and other microbial strains, offering exciting potential for applications in food preservation and beyond.

## 1. Introduction

Antimicrobial peptides (AMPs), derived from various protein-rich by-products through enzymatic hydrolysis, provide a promising alternative to synthetic additives and antibiotics in the food industry [1]. These peptides not only contribute to cost-effective waste disposal but also enhance the value of products by serving as the foundation for innovative processes within the context of a circular economy [2,3]. A notable source of such peptides was found in milk proteins within dairy white wastewater (WW). Indeed, white wastewater, a by-product of dairy processing and cleaning-in-place, inherently contains residual milk proteins, primarily casein and whey proteins [4,5]. In addition, a previous study by Damen et al. (2024) [5] regarding antimicrobial activities from milk proteins in WW highlighted that enzymatic hydrolysis of these proteins emerges as a pivotal process to produce several known AMPs and other potential antifungal peptides after 4 h of hydrolysis with Pronase E.

Nevertheless, following enzymatic hydrolysis, the presence of other non-bioactive peptides tends to dilute the overall activity of the raw hydrolysate, which represents a complex mixture of peptides; both the concentration of peptides along with potential peptide-peptide interactions contribute to the reduction in activity [6]. As a result, a necessity arises to concentrate and/or enhance the observed activity within protein hydrolysates from food by-products, aiming to generate concentrated products rich in peptides of interest, such as antifungal peptides, which remain underexplored. In the realm of peptide concentration processes, various membrane technologies have been employed for fractionating and purifying peptides from hydrolysates, such as ultrafiltration (UF) and nanofiltration (NF), that have garnered significant attention due to their cost-effectiveness and easily scalable parameters [7,8]. Despite being the most widely used methods, these technologies have exhibited limitations, with selectivity (impossible to separate peptides of the same MW but different charges) and membrane fouling emerging as significant challenges. Alternatively, electromembrane processes offer an avenue for peptide concentration. Mainly, the electrodialysis with filtration membrane process is of paramount importance in valorizing hydrolysates of dairy wastewater proteins due to its ability to selectively separate peptides based on charge and molecular masses [2]. Subjected to an electric field, cationic and anionic peptides undergo migration towards either the cathode or anode, respectively, and their potential migration is determined by the molecular weight cut-off of the filtration membrane stacked in the ED cell [2]. This electrically driven mass transfer enhances the efficiency of this separation process [6]. The combination of electrodialysis with filtration membranes has shown potential applications for the separation and recovery of bioactive compounds from food hydrolysates and the isolation of polyphenols from different sources [2,9].

Despite growing interest in dairy wastewater valorization, existing studies have focused primarily on nutrient removal, biogas production, or water treatment. Few have explored the targeted recovery of functional bioactive peptides, especially those with antifungal activity, from WW. Furthermore, limited research has assessed how separation processes such as electrodialysis with ultrafiltration membranes can enhance peptide bioactivity through physicochemical selectivity. Hence, in the pursuit of eco-efficiency and the development of new value-added products in a circular economy, this study aims to achieve the following objectives: (1) to concurrently separate specific peptide fractions based on their net charges and molecular weight from WW milk proteins hydrolysate using EDUF, (2) to identify peptides and their key characteristics in the separated peptide fractions, (3) to study the performances of the process in terms of migration rates, relative energy consumption (REC), fouling and membrane integrity, and (4) to assess the effectiveness of these fractions against bacterial and fungal strains.

## 2. Materials and Methods

### 2.1. Materials

#### 2.1.1. Chemicals

Hydrochloric acid (HCl, 0.1N) and sodium hydroxide (NaOH, 0.1N) solutions were acquired from Fischer Scientific (Montréal, QC, Canada) for the cleaning of the electrodialysis system. Potassium chloride (KCl 2 g.L^−1^) was obtained from EMD Chemicals Inc. (Port Wentworth, GA, USA), and sodium sulfate (Na_2_SO_4_) was provided by ACP Inc. (Montréal, QC, Canada).

#### 2.1.2. White Wastewater Hydrolysate

The white wastewater (WW) used in this study was produced during the initial rinsing phase of a clean-in-place (CIP) cycle of an industrial ultrafiltration system for skimmed milk. This WW was provided by Lactalis Canada (St-Hyacinthe, QC, Canada). The detailed composition of the WW was determined and previously described in Damen et al. [5]: total solids, 1.38 g/100 g dry basis; ash, 0.17%, humidity, 98.62%; fat, 0.01%, protein, 1.28%; calcium 83.3 ppm; potassium, 1.9 ppm; magnesium, 4.2 ppm; sodium, 6.1 ppm and phosphorus, 46.9 ppm. As the focus was on peptide recovery and characterization, the composition of the WW after enzymatic hydrolysis and EDUF treatment was not determined in terms of global physicochemical properties. Instead, peptide concentration, molecular mass, charge distribution, and antifungal bioactivity were assessed as indicators of compositional change relevant to the study objectives.

Briefly, the white wastewater (WW) hydrolysate used in the EDUF process was obtained through hydrolysis using Pronase E from *Streptomyces griseus*, following the same protocol outlined by Damen et al. [5]. In brief, 600 mL of WW with a protein content of 1.28% (*w*/*v*) was subjected to enzymatic hydrolysis at pH 7.5 for 4 h, with continuous agitation and controlled temperature and pH. No pretreatment was performed on the WW before enzymatic hydrolysis. Samples were taken at various time intervals, and hydrolysis was terminated by heating for 15 min at 85 °C. The resulting hydrolysates, produced in 3 separate batches, were lyophilized and stored shielded from light at −20 °C for further analyses, including determining the degree of hydrolysis and identifying peptide sequences. For peptide identification, samples were filtered using 0.45 µm PVDF filters prior to UPLC-MS/MS analysis to remove particles and ensure compatibility with the chromatographic system.

#### 2.1.3. Membrane Materials

Food-grade Neosepta cation-exchange (CEMs: CMX-FG) and anion-exchange (AEMs: AMX-FG) membranes, commercially sourced, were acquired from Astom (Tokyo, Japan). The polyethersulfone (PES) filtration membrane (FM) (commercial membrane code: MQ) with a molecular weight cut-off of 50 kDa, obtained from Synder (Vacaville, CA, USA) was employed. This FM was selected since previously, on whey protein hydrolysate, this FM was reported to yield a higher peptide concentration in the recovery compartment [10]. Importantly, this effect was achieved without significant impacts on selectivity when compared to lower cut-offs and with a reduced fouling propensity [10,11]. The main physicochemical characteristics of the PES-50 kDa are contact angle, 79.2°; volumetric porosity, 0.52 cm^3^/cm^3^; zeta potential, −13.4 mV; Rz, 9.04 μm [10]. In addition, the 50 kDa molecular weight cut-off was chosen, based on previous research on electrodialysis with filtration membranes, as well as electromembrane processes in general, demonstrating that the molecular weight cut-off of a membrane should be substantially higher—at least ten times greater than that of the target molecule to facilitate its optimal migration [8,10]. In addition, it is important to mention that before use, all membranes (CEM, AEM, and FM) were stored in a 2 g/L KCl solution to ensure a good electrical conductivity, mainly for the filtration membrane.

#### 2.1.4. Electrodialysis Configuration

**Figure 1:**
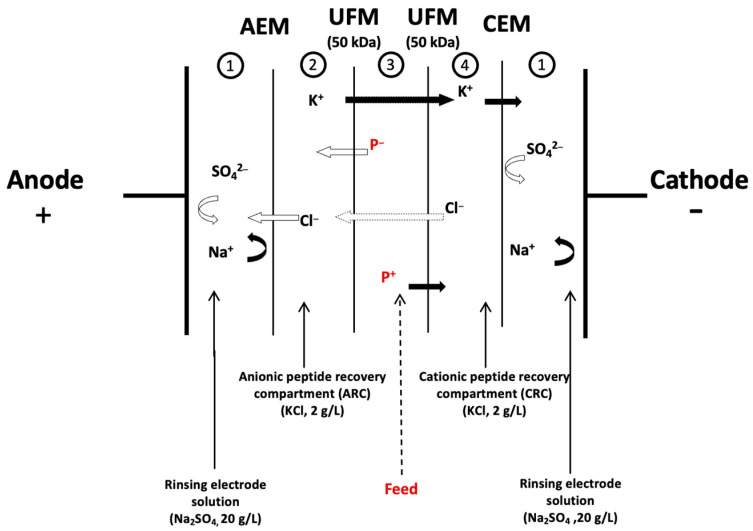
Schematic representation of the electrodialysis with ultrafiltration membrane configuration for simultaneous recovery of cationic and anionic peptides. CEM: cation-exchange membrane; AEM: anion-exchange membrane; UFM: ultrafiltration membrane; P^+^: positively charged peptides; P^−^: negatively charged peptides.

The electrodialysis system employed in this study was an MP-type cell (Electrocell Systems AB, Täby, Sweden) with an effective surface area of 100 cm^2^. The anode consisted of a DSA O2 (titanium anode), and the cathode was made of stainless steel, both sourced from Electrocell Systems AB Company (Täby, Sweden). To facilitate the recovery of both positively and negatively charged peptides, a double configuration was adopted. The cell was constructed by stacking one AEM, two UFMs of 50 kDa, and one CEM [10] (Figure 1). This electrodialysis (ED) cell was partitioned into five compartments, forming four distinct closed loops that were interconnected to four external tanks, enabling continuous recirculation. The recirculation process was driven by four centrifugal pumps (Baldor Electric Company, Fort Smith, AR, USA). In the first loop (comprising compartments 1), an electrode-rinsing solution containing 800 mL of Na_2_SO_4_ circulated at a flow rate of 1200 mL.min^−1^. Subsequently, compartments 2 and 4 received a 600 mL KCl at 700 mL.min^−1^ each, intended for the recovery of anionic and cationic peptides, respectively. Compartment 2 (ARC) recovered the anionic peptides, while compartment 4 (CRC) was designated for the recovery of cationic peptides. Finally, compartment 3 housed the feed solution of white wastewater hydrolysate at 1.28% (*w*/*v*) of proteins, with a flow rate of 700 mL.min^−1^.

### 2.2. Protocol

Initial assessments, aimed at determining the limiting current density (LCD) following the Cowan and Brown method [12]. Under the actual treatment conditions and with the specified configuration (Figure 1), the LCD calculated was corresponding to 55 A/m^2^ at a voltage of 8.5 V and a current intensity of 0.5 A. Due to the relatively low limiting current densities, and following this determination, the applied voltage was set at 7.8 V, corresponding to an electrical field strength of 2 V/cm between the electrodes to compare with other previous studies [6,13]. Voltage monitoring was carried out using a multimeter directly connected to the electrodes, with the voltage difference generated by a 0–30 V power supply (HQ Power PS3003, Xantrex, Burnaby, BC, Canada). The flow streams of the solutions remained unchanged throughout the process. The conductivity of KCl solutions (ARC and CRC) was initially matched to the feed and increased progressively in parallel during the process to ensure the continuous and linear migration of peptides [14]. The pH of the feed at 7.5 following the enzymatic hydrolysis was maintained constant throughout the process by adding NaOH (0.1 N) solution. The experimental parameters (pH and conductivity) were measured every 10 min during the treatment. The duration of the EDUF was 180 min [6,10]. In total, 5 mL of samples from the hydrolysate and each recovery compartment (feed, anionic, and cationic) were collected before applying voltage and during the treatments at 0, 30, 60, 120, and 180 min. After EDUF (180 min), the recovered fractions from their respective compartments were freeze-dried and stored at 4 °C until further analyses. Three independent EDUF experiments were carried out, and a new set of membranes was used for each experiment to ensure repeatability.

### 2.3. Analyses

#### 2.3.1. Conductivity and pH Measurements

A YSI conductivity meter (Model 3100) equipped with a YSI immersion probe model 3252 (cell constant K = 1 cm^−1^, Yellow Springs Instruments Co., Yellowsprings, OH, USA) was used to measure the solution conductivity in compartments 2, 3, and 4 (Figure 1). pH levels were also monitored in the same compartments utilizing pH meters Orion Star A221 (Thermo Scientific, Montreal, QC, Canada).

#### 2.3.2. Protein/Peptide Concentrations

For the protein/peptide concentration, the total nitrogen content of the recovery compartment solutions was determined on freeze-dried samples using a rapid MicroN cube (Elementar, Langenselbold, Germany) based on the Dumas method. The protein/peptide concentration was estimated with a conversion factor of 6.38 [15].

Total peptide concentrations in anionic, cationic, and feed compartments were determined using µBCA protein assay from the samples withdrawn over a period of 180 min. This method employs a colorimetric approach for peptide quantification, relying on the reaction with bicinchoninic acid (BCA), which is particularly effective for dilute protein samples [6]. Bovine serum albumin standards were utilized to establish a standard curve following the manufacturer’s recommendations. In the assays, 150 µL of either the standards or the samples was combined with 150 µL of the working reagents in a microplate, followed by a 2-h incubation at 37 °C. Absorbance readings were taken at 562 nm using the xMark Microplate spectrophotometer from Bio-Rad (Mississauga, ON, Canada) and then converted into protein concentrations (µg/mL).

#### 2.3.3. Peptide Recovery Yield

The initial hydrolysate was found to contain 1.28% peptides based on nitrogen content (Section 2.3.2.). The peptide recovery yield over time was determined by measuring the concentration of peptides in the anionic and cationic recovery fractions using a μBCA assay (Section 2.3.2.). The yield was calculated with the following Equation (1) for both fractions:

(1) $$\mathrm{Peptide}\mathrm{recovery}\mathrm{yield}\left(\%\right)=\frac{C_{t}}{C_{initial}}\times 100$$

where *C_t_* represents the peptide concentration in the recovery compartment (g BSA equivalent/mL), and C_initial_ denotes the peptide concentration in the initial hydrolysate (g BSA equivalent/mL).

#### 2.3.4. Migration Rate

The global migration rate (MR) of peptides was determined by calculating the total quantity of peptides in grams, derived from the total nitrogen content assessed through the Dumas method (Section 2.3.1). Initially, the MR was computed based on the overall process duration using Equation (2) [10]:

(2) $$MR=\frac{m_{peptides}}{S\times T_{process}}$$

Here, MR is expressed in g/m^2^.h, m_peptides_ the quantity of peptides recovered in gram (g), S denotes the ultrafiltration membrane surface (0.01 m^2^), and T_process_ represents the duration in hours (h) of the separation process.

#### 2.3.5. Relative Energy Consumption and Global Resistance

The relative energy consumption (REC) was determined using Equation (3) [10]:

(3) $$REC=\int_{0}^{t}\frac{U\cdot I\cdot (t)}{m_{peptides}}dt$$

where REC is expressed in Wh/g of peptides, U the voltage in volts (V), I the current intensity in amperes (A), t the duration in seconds (s) and m_peptides_ the quantity of peptides recovered in grams (g).

The global resistance was determined using Ohm’s law (U = RI). Voltage (V) and current (A) values were continuously monitored directly from the power supply.

#### 2.3.6. Thickness and Electrical Conductivity of Membranes

The thickness and conductance of each membrane were assessed both before and after the EDUF run to determine their electrical conductivity and evaluate potential changes in the membrane integrity. The membrane thickness was measured using an electronic digital micrometer from Marathon Watch Company LTD (Richmond Hill, ON, Canada). Prior to analyses, the cation-exchange (CEMs), anion-exchange (AEMs), and ultrafiltration membranes (UFMs) were soaked in 0.5 M NaCl for 30 min. All membranes were stored in deionized water at 4 °C between uses. Conductance measurements were conducted with a YSI conductivity meter (Model 3100). The meter was connected to a specially designed clip provided by Institut de Chimie et des Matériaux Paris-Est (ICMPE) at Université Paris-Est in Thiais (Val de Marne, France). Before each measurement, the membranes were immersed for equilibration in a 0.5M NaCl solution for 30 min [10].

Following the measurements, membrane conductivity was calculated using Equation (4) [16]:

(4) $$\kappa =\frac{l}{R_{m}\times A}$$

where κ is the membrane conductivity (mS/cm), l is the membrane thickness (cm), A is the electrode area, which is 1 cm^2^, and R_m_ is the membrane resistance (Ω) determined using the following Equation (5) [17]:

(5) Rm=1Gm=1Gm+s−1Gs=Rm+s−Rs

where G is the conductance (in S), m represents the membrane, m + s the membrane in the reference solution of NaCl, and s the reference solution.

#### 2.3.7. Peptides Characterization by UPLC-MS/MS Analysis

UPLC-MS-MS-QTOF analyses were carried out using the same 1290 Infinity II UPLC system (Agilent Technologies, Santa Clara, CA, USA) as described by Damen et al. [5]. Fractions collected from the feed and recovery compartments after EDUF samples were filtered through a 0.45 µm PVDF filter into glass vials, with 10 µL loaded onto an InfinityLab Poroshell 120 EC-C18 column (2.1 × 100 mm, 2.7 micron, Agilent, CA, USA). The column operated at a flow rate of 0.4 mL/min at 45 °C, with a maximum pressure of 600 bar. Prior to initiating the gradient, the column was equilibrated at 1% B with mobile phases of solvent A (LC-MS grade water with 0.1% formic acid) and solvent B (LC-MS grade ACN with 0.1% formic acid). The gradient started with a ramp to 45% B over 30 min, followed by a cleaning step at 95% B for 5 min, and finally a re-equilibration to the initial conditions for an additional 5 min before the next injection. Peptide sequences in the hydrolysates were identified using a hybrid ion mobility quadrupole time-of-flight mass spectrometer (6560 high-definition mass spectrometry (IM-Q-TOF), Agilent, CA, USA). All LC-MS/MS experiments utilized Q-TOF, with signals recorded in positive mode at Extended Dynamic Range, 2 GHz, 3200 *m*/*z*, and a scan range from 100 to 3200 *m*/*z*. Nitrogen served as the drying gas at 13.0 L/min and 150 °C, and as the nebulizer gas at 30 psi. The capillary voltage was set at 3500 V, the nozzle voltage at 300 V, and the fragmentor at 400 V. Calibration of the system was carried out using an ESI-L low concentration tuning mix (Agilent Technologies, Santa Clara, CA, USA). Data acquisition and analysis were completed using Agilent Mass Hunter Software (LC/MS Data Acquisition, Version B.09.00, Qualitative Analysis, Version B.07.00 Service Pack 2 with BioConfirm Software v. B.07.00).

#### 2.3.8. In Silico Bioinformatic Analyses

The structural and physicochemical properties of identified peptides were analyzed using bioinformatics resources to pinpoint peptides with potential for antimicrobial activities. Several databases, including APD3 (https://aps.unmc.edu/, 9 June 2023), DBAASP (https://dbaasp.org/search, 10 June 2023), BioPepdb (http://bis.zju.edu.cn/biopepdbr/, 9 June 2023), Peplab (https://www.pep-lab.info/, 9 June 2023), DFBP (http://www.cqudfbp.net/, 10 June 2023), CAMPr4 (https://camp.bicnirrh.res.in, 9 June 2023) and DbAMP (https://awi.cuhk.edu.cn/dbAMP/, 10 June 2023), were employed to elucidate critical physicochemical parameters influencing the peptides’ antimicrobial activities. This comprehensive analysis, which included details on the peptides’ chemical structure, physico-chemical properties (molecular mass, peptide length, isoelectric point (pI), GRAVY Score, and net charge at pH 7.0), and experimentally tested activities, was crucial for assessing their bioactive potential.

#### 2.3.9. Antimicrobial Assays

The initial hydrolysate, as well as the fractions collected from the feed, ARC, and CRC after 180 min of EDUF, were tested. The antimicrobial assays (agar well diffusion assay and minimal inhibitory concentration) were conducted to assess the antimicrobial activity of the anionic, cationic and the final recovery feed obtained compared to the initial hydrolysate. The antimicrobial activities were tested against two bacterial strains, *Clostridium tyrobutyricum* (ATCC25755) and *Pseudomonas aeruginosa* (ATCC15442), and two fungal strains, *Mucor racemosus* (LMA722) and *Penicillium commune* (LMA72). These species were chosen since they are often responsible for the alteration of shredded cheese, such as cheddar and mozzarella, and to compare with a previous study [5,18]. The antimicrobial activities of the different samples were evaluated using an agar diffusion method as described by Damen et al. [5]. The tests were conducted on Tryptic Soy Agar (TSA) for bacteria and Yeast Extract Glucose (YEG) Agar (Sigma-Aldrich, Saint-Louis, MO, USA) for molds. Specific inoculum preparations and concentrations were used, and hydrolysate samples were tested at 40 mg/mL of peptides [3,5]. Appropriate controls were included, and the guidelines of the Clinical and Laboratory Standards Institute were followed, with results measured in triplicate to ensure accuracy and reproducibility [19].

The minimum inhibitory concentration (MIC) of the different fractions was also determined using the protocol by the European Committee for Antimicrobial Susceptibility Testing [20]. The MIC assays were conducted using Potato Dextrose broth (PDB) (BD-Difco, Sparks, MD, USA), containing a known peptide concentration. The initial peptide concentration tested was 40 mg/mL, which was serially diluted in PDB in the microplate [5,21]. Bacterial inoculum was prepared to achieve a concentration of 1 × 10^6^ CFU/mL; however, mold spore inoculum was prepared to reach a concentration of 1 × 10^3^ CFU/mL in each well. The microplates were incubated for 24 h at 37 °C for *C. tyrobutyricum* and *P. aeruginosa* and at 25 °C for *M. racemosus* and *P. commune*, and a microplate spectrophotometer was used for quantification, based on absorbance measurements (Infinite^®^ F200 PRO, Tecan Inc., Durham, NC, USA). The MIC was defined as the lowest peptide concentration entirely inhibiting microbial growth, assessed by measuring absorbance at 595 nm. Positive controls for the MIC assays consist of ampicillin (256 mM) for bacteria and natamycin (16.7 µg/mL) for molds (Sigma-Aldrich, Saint-Quentin Fallavier, France) [5]. Also, negative controls included culture media and media with the tested sample to account for intrinsic absorbance, ensuring accurate MIC determination.

#### 2.3.10. Statistical Analyses

##### UPLC-MS/MS Data Treatment

The recovered fractions were analyzed using the Agilent Mass Hunter Software package (Agilent Technologies, Santa Clara, CA, USA), with MS spectra and UV chromatograms acquired using LC/MS Data Acquisition Version B.08.00. This method is consistent with the approach applied in the previous study by Damen et al. [5]. This analysis aimed to identify key peptides that represented the overall hydrolysis of milk proteins under various conditions. The MS spectra were compared with known milk protein sequences (UniProtKB—P02666, P02668, P02662, P02663, P00714, P02754) to determine the sequence, molecular mass (Da), and specific location of each identified peptide on the protein sequence. To ensure reliability, only peptides detected in at least two out of three replicates were considered [6]. Statistical analysis of ion abundances was conducted using Mass Profiler Professional Software version 15.1 (Agilent Technologies, Santa Clara, CA, USA), following the procedure and statistical methods outlined by Cournoyer et al. (2024) [4]. Peptides were filtered by frequency with a cut-off of 100%. To compare ion abundances between conditions for each peptide, a non-parametric Kruskal–Wallis test was applied, chosen for its robustness given the lack of strong support for normality and homogeneity of variance in the data [6]. The Kruskal–Wallis test is advantageous as it does not assume a specific data distribution [21]. To control the rate of Type I errors, the false discovery rate (FDR) method, specifically the Benjamini–Hochberg procedure, was used [22]. This method, along with a permutation resampling approach involving 10,000 permutations, helped ensure that the identified significant peptides were not due to random chance. Significant peptides were then subjected to a multiple comparison Tukey HSD test [23], which allowed pairwise comparisons of conditions to identify statistically significant differences [6]. This test is particularly effective in comparing means across multiple groups while controlling the overall Type I error rate. Finally, a hierarchical cluster heatmap was performed on the remaining peptides to examine potential groups of peptides in different recovery compartments and to study the general behavior of the separation process by EDUF.

##### Other Statistical Analyses

All experiments were conducted using three separate WW protein hydrolysates. The results are presented as mean values with standard deviations. Statistical analyses were performed using SigmaPlot v. 15.0 (Systat Software, Inc., San Jose, CA, USA). To assess the variation in EDUF parameters over the course of the process (conductivity, pH, global system resistance, peptide migration), regression tests were applied. For the characterization of CEM, AEM, and UFM membranes before and after the EDUF process, a paired *t*-test was used (*p* < 0.05). Additionally, the UFM membranes were evaluated using a one-way ANOVA, followed by a Tukey HSD test for mean comparison (*p* < 0.05). A detailed statistical summary for all major analyses is provided in Table 1. This includes the statistical test used, the associated F or t-values, degrees of freedom (df), and *p*-values.

## 3. Results and Discussion

### 3.1. Electrodialysis: Process Performance Evaluation

#### 3.1.1. Progression of Conductivity and pH in ARC and CRC

**Figure 2:**
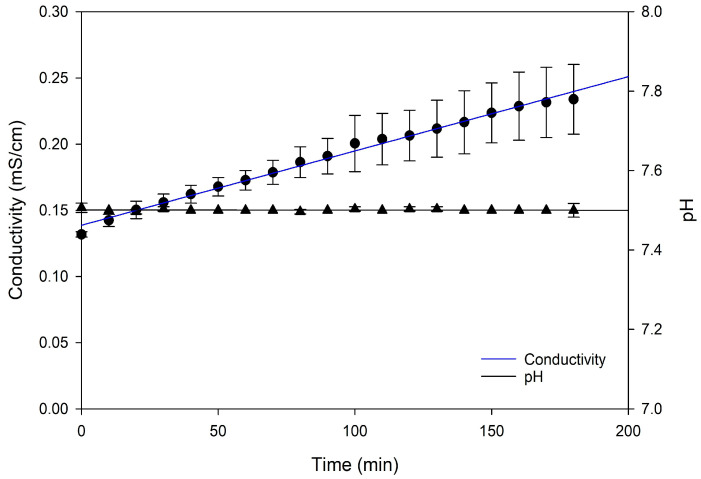
Evolution of conductivity and pH in the feed compartment as a function of time.

The evolution of conductivity and pH was monitored over a 180-min period (Figure 2). Conductivity exhibited a steady increase from an initial value of approximately 0.13 mS/cm, reaching 0.25 mS/cm by the end of the experiment. The constant and linear rise in conductivity (R^2^ = 0.9909) suggests a continuous accumulation and migration of ions between the different compartments. This ion migration likely led to higher concentrations of K^+^ and Cl^−^ ions in the feed compartment, and it was supported by the EDUF cell configuration (Figure 1), where Cl^−^ migrates through the UFM to the ARC and passes through the AEM and electrolyte, while K^+^ moves from the UFM to the CRC and CEM, eventually entering the electrolyte. Consequently, this migration of ions contributed to an increase in ion concentration in the feed, thereby elevating its conductivity. On the other hand, the conductivity of the KCl solution in the ARC and CRC dropped as soon as the voltage was applied. However, this decrease was offset by adding KCl to maintain conductivity levels equivalent to the feed during the whole process, as mentioned in Section 2.2, leading to no statistically significant difference (*p* > 0.05). The absence of a significant difference was supported by the regression results shown in Table 1. Effectively, controlling the conductivity of solutions during EDUF was essential for optimizing peptide migration because it directly influenced the movement of peptides through the filtration membrane, affecting both the efficiency and linearity of the separation process. Indeed, in their study, Suwal et al. [14] demonstrated that maintaining a constant conductivity was essential for optimizing the recovery of valuable peptides from marine protein hydrolysates during electrodialysis and that in a linear way. The study also revealed that consistent ionic strength minimized electrostatic interactions between peptides and the ultrafiltration membrane, enhancing peptide migration. Overall, conductivity could impact peptide migration with higher relative abundance in the initial hydrolysate, leading to increased migration [10,24].

As for pH, it remained stable throughout the process, consistently staying around 7.4 over a 180-min period across all compartments, including the feed, CRC, and ARC, where the trend closely followed the feed’s pH evolution by adding NaOH solution (0.1 M) (Figure 2). Crucially, maintaining a stable pH in the feed solution was essential for peptide electro-separation by EDUF, as it significantly affects the charge of amino acids and peptides and their subsequent migration during EDUF [6,25]. In addition, at lower pH levels, peptides often become more positively charged, which enhances their electrostatic attraction to negatively charged membranes, which can trigger different interaction mechanisms compared to neutral pH, including increased hydrophobicity and α-helical conformations, ultimately leading to varied effects on membrane permeability [26]. Also, pH could influence the activity of pH-sensitive peptides by altering their structures and the charge state of amino acids and functional groups, thereby affecting their physicochemical properties and bioactivities [3].

#### 3.1.2. Protein/Peptide Concentrations, Yields, and Migration Rates in the Recovery Compartments

**Figure 3:**
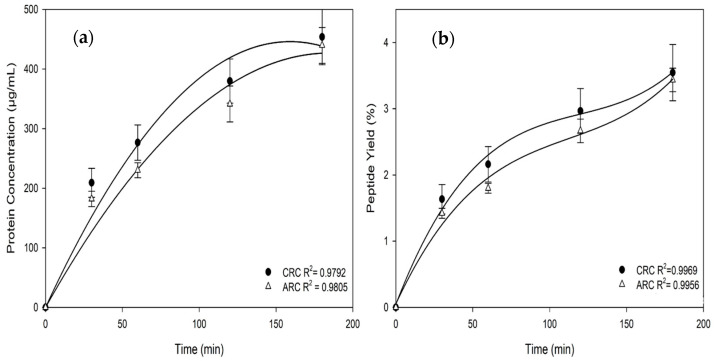
Time-dependent changes in protein/peptide concentration (**a**) and peptide yields (**b**) in the cationic recovery compartment (CRC) and anionic recovery compartment (ARC) during the EDUF Process.

Using μBCA results, protein/peptide concentrations and recovery yields were determined over the course of the experiments in both CRC and ARC (Figure 3). In the cationic recovery compartment (CRC), protein concentration increased from 0 µg/mL at the beginning to nearly 450 µg/mL at 180 min (Figure 3a), with a final peptide yield exceeding 3.5% (Figure 3b). Also, the anionic recovery compartment (ARC) exhibited a slightly lower protein concentration, rising from around 0 µg/mL to about 440 µg/mL over the same period (Figure 3a), with a final peptide yield just below 3.4% (Figure 3b). Based on the initial protein content of 1.28% (*w*/*v*) in the WW and a protein content Dumas-based estimate of 80%, the initial peptide concentration was 10.24 g/L. With approximately 20% of these peptides recovered by EDFM, this corresponds to an absolute peptide recovery of 2.0 g per liter of WW. At the reported relative energy consumption (REC) of 2.05 Wh/g, the process required approximately 4.2 Wh per liter of WW treated. This pairing of REC with throughput provides a more comprehensive view of the system’s energy efficiency. The CRC consistently showed slightly higher performance than the ARC, which could be attributed to the initial hydrolysate’s richness in cationic peptide sequences [5]. These values are slightly higher than those reported by Kadel et al. (2019), who observed migration rates of 5.10 ± 0.32 g/m^2^·h (CRC) and 3.19 ± 0.11 g/m^2^·h (ARC) using a 50 kDa UFM on whey protein hydrolysate at 0.75% *w*/*v* over 120 min [10]. The higher rates in the present study likely stem from a higher initial peptide concentration (1.28% *w*/*v*) and longer separation time (180 min). These observations were supported by regression analyses for peptide concentration and yield (Table 1), where linear and cubic models showed R^2^ values, confirming consistent peptide migration patterns. The high R^2^ values for both compartments (CRC: 0.9792 for concentration, 0.9969 for yield; ARC: 0.9805 for concentration, 0.9956 for yield) confirmed the success of the EDUF separation process. In the present study, final peptide migration rates of 6.83 ± 0.59 g/m^2^·h for CRC and 6.19 ± 0.66 g/m^2^·h for ARC were obtained whereas some authors obtained migration rates of 5.10 ± 0.32 g/m^2^·h for CRC and 3.19 ± 0.11 g/m^2^·h for ARC after 120 min of EDUF separation at 0.75% (*w*/*v*) of whey protein hydrolysate with the same EDUF configuration, same 50 kDa UFM but a shorter duration (120 min) [10]. The statistical significance of these migration rates was confirmed through quadratic regression (F = 54.45, *p* = 0.0180; Table 1). The superior performance in the present study could be attributed to the higher initial peptide concentration of 1.28% (*w*/*v*), as opposed to 0.75% (*w*/*v*) used by Kadel et al. [10]: this increased concentration allows more peptides to migrate across the membrane. Additionally, here, the EDUF treatment conditions were chosen based on previous studies specifically for peptides separation, further improving the efficiency and yield of peptide migration compared to the previous study, which utilized a shorter duration of 120 min. However, other authors obtained higher migration rates up to 8.9 g/m^2^·h for lactoferrin [27], 41.0 g/m^2^.h, 29.1 g/m^2^·h for BSA [27] for β-lg [28] in model whey or whey-enriched solutions with UFM 500 kDa and 89 kDa MWCO, respectively.

This finding was effectively linked to the work of Kadel et al. [10], who discussed the influence of electrostatic interactions between UFM and molecules. In 2021, Kadel et al. [13] also highlighted that the surface charge and roughness of membranes significantly affected electrostatic interactions with peptides, which, in turn, impacted their migration during the ultrafiltration process. In their studies, the authors used polyethersulfone (PES) membranes, as in the present work, characterized by lower peak-to-valley roughness values (Rz of 9.04 μm for the membrane used in the present study), which were demonstrated to significantly influence the migration of peptides within the electrodialysis with filtration membrane system. In addition, they demonstrated that the negatively charged surface of the PES membranes (Zeta potential of −13.4 mV for the membrane used in the present study) was found to attract positively charged peptides, thus enhancing their recovery in the CRC. Conversely, the electrostatic interactions impeded the migration of anionic peptides in the ARC, due to electrostatic repulsion with the membrane. Despite these differing interactions, the experimental setup and operational conditions were adjusted such that both compartments ultimately achieved the same concentration and yield of peptides, indicating a balanced optimization of the electrodialysis process for both types of peptides [25]. This was also consistent with the findings of Henaux et al. [29], who reported that cationic peptides exhibited better recovery rates due to favorable interactions with the membrane surface during ultrafiltration (50, 20, and 5 kDa MWCO), and Geoffroy et al. [24], who highlighted that a CEM/UFM/AEM configuration led to a 26.5% reduction in environmental impact costs and an 18% increase in the recovery of bioactive cationic peptides. Overall, the peptide migration rates observed here align well with the trend reported by Geoffroy et al. (2022), who demonstrated that optimization of configuration and concentration can significantly enhance EDUF efficiency and peptide recovery [24].

#### 3.1.3. Global Resistance and REC

**Figure 4:**
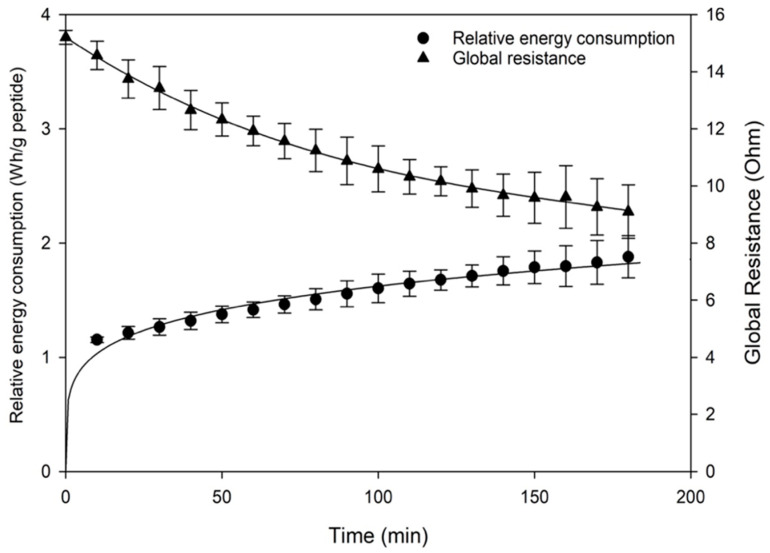
Global resistance and relative energy consumption (REC) evolution during the EDUF process as a function of time.

Regarding global resistance, a reduction in resistance from 15.2 Ω to 9.1 Ω was observed after three hours, as depicted in Figure 4, and was consistent with observations reported in the literature on conventional ED [29]. This trend was statistically significant as confirmed by quadratic regression (Table 1). This is consistent with findings from conventional ED studies, such as Geoffroy et al. (2022), where reduced resistance was associated with increased ion availability and conductivity, improving separation performance [24]. However, Cournoyer et al. [6], who studied the impact of different current modes on the migration of porcine cruor hydrolysate peptides during EDUF, observed a surprising increase in the global resistance after 180 min in continuous current while no fouling was detected. In the present study, the drop in resistance was likely due to the linear increase in conductivity in the feed compartment due to the addition of KCl salt in both recovery compartments to support ongoing ion migration. In electrodialysis, ions are driven through selective membranes by an electric field: a higher ion concentration in the solution enhances conductivity, enabling more efficient ion migration and subsequently lowering electrical resistance [10,24]. This interplay between ion concentration, conductivity, and resistance underscores the importance of managing these factors to optimize conventional electrodialysis efficiency [30]. The UFM membranes also likely contributed to reduced resistance and fouling due to their previously discussed electrostatic properties (see Section 3.1.2), which supported continuous peptide migration during the process. As a result, with increased ion concentration and solution conductivity, the global system resistance diminished, enhancing the efficiency of the electrodialysis process, especially since no fouling was observed on the UF membranes.

The increase in relative energy consumption (REC) observed in this study, from 1.15 Wh/g peptides to 2.05 Wh/g peptides over three hours of EDUF, was contextualized by comparing it with findings from recent research on EDUF energy use. For instance, Cournoyer et al. [6] reported a significantly higher REC of 39.76 ± 4.77 Wh/g peptides when continuous current was applied for peptide migration from porcine cruor hydrolysate. This highlights the comparatively lower energy cost of the current EDUF setup, which benefits from optimized voltage, membrane selection, and peptide concentration. Similarly, Geoffroy et al. (2022) showed that tailoring EDUF parameters could reduce environmental impact while improving yield and energy efficiency [24]. This suggested that, although the REC values in the current study were lower, energy demands in continuous current electrodialysis varied greatly depending on the specific process conditions and configurations used. Other studies also obtained an increase in REC from 7.3 to 13.9 Wh/g peptide of a whey protein hydrolysate with a cell configuration CEM/UFM/CEM at a semi-industrial scale (1.2 A, 3 h duration, and 20 kDa) [24].

#### 3.1.4. Membrane Characterization

The characterization of the membranes used in the process could be pivotal in determining the efficiency of peptide recovery, as it directly influences fouling behavior and overall process effectiveness. Therefore, the thickness and electrical conductivity of all three membrane sets were measured before and after the EDUF process, and the average values for both parameters were calculated for each type of membrane (Table 2).

The analysis of membrane characteristics indicated that membrane thickness remained unchanged during the EDUF process, with a *t*-test showing no significant difference (*p* > 0.05; Table 1), suggesting that the process did not affect the structural integrity of the membranes. The conductivity results for ion exchange membranes showed statistically significant reductions, with *p*-values well below the threshold of 0.05 (Table 1). The AEM and CEM membranes exhibited reductions in conductivity of about 18.3% and 18.1%, respectively, but the changes were below 20% which was considered low to potentially influence their performance and have consequences on the process [31]. In the initial hydrolysate, the presence of divalent ions like calcium and magnesium may have substantially lowered the conductivity of ion exchange membranes. Divalent ions exhibit reduced mobility compared to monovalent ions due to their larger ionic radius and the stronger electrostatic interactions they engage in with the surrounding medium [32]. This decreased mobility could lead to a reduction in the conductivity of the system, as the overall ionic transport is hindered [32]. Also, this reduction was largely attributed to the robust attachment of these ions to the functional groups on the membranes [33,34,35,36]. Such bindings decreased the number of available charge carriers, limited the dissociation of these functional groups, and modified the microstructure of the membranes. These changes might influence membrane performance and may be related to minor fouling effects, where positively and negatively charged peptides migrate towards the cathode and anode, respectively, and are unable to pass through the membrane [29]. Also, the adsorption of peptides onto the membrane surface, due to electrostatic interactions with membranes, further exacerbated the reduction in conductivity [24,29,37].

Conversely, UFM did not show any significant differences in conductivity for UFM 1 and UFM 2, suggesting stable ionic migration performance. The Tukey HSD test confirmed no significant differences between UFM 1 and UFM 2 (*p* < 0.05).

As discussed in Section 3.1.2. and in previous studies [10,11], the UFM membranes’ characteristics contributed to maintaining their conductivity, which supported continuous peptide migration during the process. However, other characteristics, such as π–π stacking interactions and contact angle, could also significantly influence the performance of these membranes, but these parameters were previously investigated by Kadel et al. [10]. Consequently, to confirm the selectivity of the EDUF process and the observed differences between the CRC and ARC, chromatography analyses were conducted to assess the peptide population and identify the migrated peptides.

### 3.2. Peptide Population Characterization

#### 3.2.1. Analyses of UV Chromatograms

**Figure 5:**
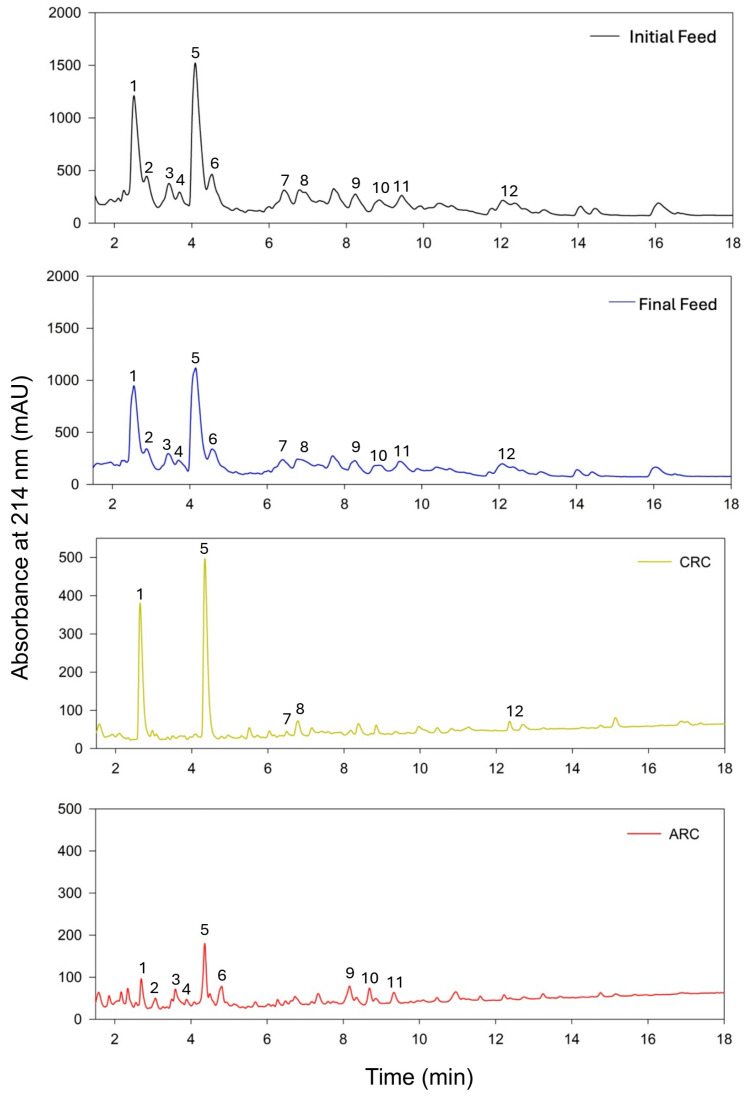
UV chromatograms of peptide separation across initial hydrolysate, final feed, cationic recovery compartment (CRC), and anionic recovery compartment (ARC) following electrodialysis with ultrafiltration (EDUF) process.

The UV chromatograms presented in Figure 5 illustrate the peptide profiles of the initial hydrolysate, feed, and fractions recovered in the CRC and ARC after the EDUF process. Peaks marked with numbers correspond to specific peptide fractions present in the initial hydrolysate, feed, and either the CRC or ARC, providing insights into the selective separation achieved by the EDUF process. The UV chromatogram of the initial hydrolysate showed a broad distribution of peptides across various retention times, indicating a heterogeneous but similar peptide population in both initial hydrolysate and feed solutions. Peaks 1, 5, and 6 were particularly prominent, with high absorbance, suggesting these peptides or amino acids were abundant in the initial hydrolysate. The final feed chromatogram closely mirrors the initial hydrolysate, but the intensity of the peaks was slightly reduced, reflecting a partial separation of peptides during the EDUF process. The presence of most peaks in both the initial hydrolysate and feed indicated that a significant portion of the peptides was not fully migrated in the separation treatment. In the CRC chromatogram, the peptide profile was notably simplified compared to the initial hydrolysate and feed. The most dominant peaks in the CRC were peaks 1 and 5. Peak 1 was present but with reduced intensity, while peaks 2, 3, and 4 were absent, confirming their selective separation into other compartments or no migration during the process. This evaluation of the chromatogram demonstrated that the CRC effectively captured some peptides, reducing the complexity of the peptide mixture and highlighting its role in concentrating specific peptides based on charge. In contrast, the ARC chromatogram showed the presence of peaks 2, 3, and 4, which were initially prominent in both the hydrolysate and feed. However, peaks 1 and 5, which dominate all the other chromatograms, were in low concentrations in the ARC, further confirming the selective partitioning of peptides between the two compartments. Also, peaks 9, 10, and 11, which were present in both the initial hydrolysate and feed chromatograms, were effectively separated into the ARC but were absent in the CRC. Overall, the ARC chromatogram showed a lower absorbance, reflecting a lower peptide concentration compared to the CRC, likely due to the relative scarcity of strongly anionic peptides in the hydrolysate.

#### 3.2.2. Hierarchical Clustering and Heatmap Visualization

**Figure 6:**
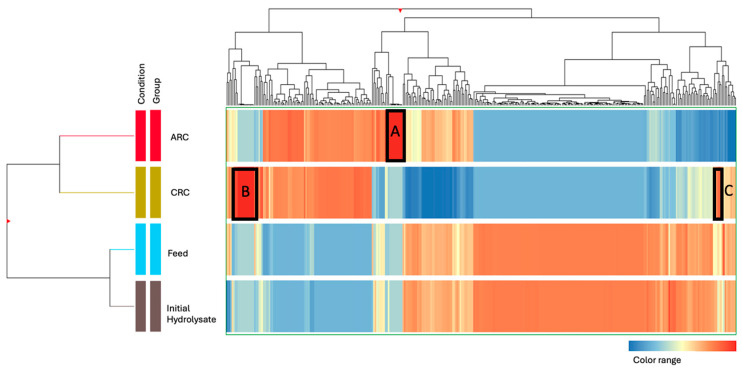
Hierarchical cluster heatmap illustrating peptide distribution and separation from initial hydrolysate efficiency across anionic, cationic, and feed compartments in electrodialysis with ultrafiltration (EDUF) after 180 min.

Following the UV chromatogram analysis, which highlighted overall differences in peptide profiles between the ARC, CRC, and feed solutions, a hierarchical cluster heatmap (Figure 6) was generated to further assess these differences at the peptide level. It was represented by averaging MS/MS ion abundances data for each peptide and then using Euclidean distance to group similar peptides together to compare the peptide population separated in each recovery compartment during the EDUF process [6]. The hierarchical clustering dendrograms on the left and top axes indicated distinct grouping of peptides based on their ion abundances following the EDUF treatment. In the ARC, the heatmap revealed a high intensity of colors in the red to yellow range, suggesting a significant concentration of peptides with potentially anionic characteristics, especially those in box A.

Conversely, the CRC was characterized by a distinct pattern with blue to yellow tones representing peptides that had been attracted to the negatively charged electrode. The regions of high peptide concentration observed in the UV chromatograms correspond to the red zones, highlighted with a black box, in the ARC (box A) and CRC (boxes B and C) sections within the heatmap, indicating a successful separation of peptides: the presence of strong peaks like peak 1 and 5 (Figure 5) in the CRC chromatogram indicates the concentration of peptides, which corresponds to the red clustering of peptides in the heatmap (box B). Similarly, the peaks in the ARC chromatogram suggest the presence of peptides, aligning with the red clustering of other different peptides in the heatmap (peaks 2, 3, 4, 6, 9, 10 and 11). The feed solution showed a more heterogeneous distribution of colors, spanning from blue to red, which suggested the presence of a broad spectrum of peptides, potentially under 50 kDa, that were not entirely separated by charge or via electrostatic or hydrophobic interaction [6,24,25]. This residual peptide mix could represent those with intermediate properties or those less effectively influenced by the electric field during the process.

#### 3.2.3. Peptide Identification and Characterization

To complement the findings from the UV chromatograms and the hierarchical cluster heatmap, a detailed analysis of the 29 peptides selected in the red zones of the heatmap (boxes A, B and C), including molecular mass, net charge at pH 7, GRAVY score, pI, and sequence data from 20 peptides in the CRC and 9 peptides in the ARC compartments corresponding to those highlighted in the red zones of the heatmap, was performed to assess the effectiveness of the separation process (Table 3). The peptides present in the significant peaks of the UV chromatograms demonstrate both the concentration and selective partitioning of peptides based on charge and size across the compartments: all of the peptides in both CRC and ARC exhibited molecular masses below 50 kDa, in line with the membrane’s cutoff, which allowed smaller peptides to pass through. The separation of peptides across EDUF compartments resulted from a complex interaction of molecular size, charge, and membrane-specific properties. The peptide distribution observed in the UV chromatograms highlights the role of both physical and chemical characteristics in achieving efficient separation and accurate quantification of peptides within complex mixtures [10]. In the CRC, these peptides predominantly displayed positive net charges at pH 7, aligning with the cationic nature of the peptides recovered in this compartment. For instance, peptide GPVRGPFP, derived from Casein β (199–206) and corresponding to peak 12 in the CRC UV chromatogram, had a molecular mass of 825.45 Da and a net positive charge of +1.00 at pH 7. Other examples include QRF (449.24 Da, +1.00, peak 5) and SRYP (521.26 Da, +0.99, peak 1), which also exhibited strong positive charges, reinforcing the cationic selectivity of the CRC. Neutral peptides such as TPV (319.16 Da, 0.00, peak 5) and LPLP (434.21 Da, 0.00, peak 12) were also recovered, likely due to their small size or interactions with other cationic peptides, such as being carried along by electrostatic or hydrophobic interactions [6,24,25]. In contrast, the ARC contained peptides that were predominantly anionic, with negative net charges at pH 7, consistent with the migration of anionic peptides towards the anode. Peptides like DEALEK derived from β lactoglobulin (130–135), corresponding to peak 4 in the ARC UV chromatogram, had a molecular mass of 703.34 Da and a net negative charge of −2.00 at pH 7, exemplifying the ARC’s ability to capture anionic species. Other examples include GDLEI (545.27 Da, −2.00, peak 9) and DEL (375.17 Da, −2.00, peak 4), which further highlight the compartment’s role in selectively recovering peptides with negative net charges. The presence of peptides with neutral or slightly positive charges, such as PQYLKT (748.36 Da, +1.00, peak 6) in the ARC, suggests that these peptides may have been separated due to their association with other anionic peptides by electrostatic or hydrophobic interactions, as indicated by their placement in the heatmap’s red zones. Interestingly, MKEGIHAQQ (1040.51 Da, peak 5) was found in both CRC and ARC compartment. This peptide was identified as a potential antifungal agent in a previous study by Damen et al. [5] through their comparative analysis between different hydrolysis enzymatic conditions. These findings suggest that neutral peptide may also possess antifungal properties, warranting further investigation through chemical synthesis to confirm and evaluate its bioactivity. Overall, the comparison between the CRC and ARC demonstrates a clear distinction in the peptides recovered by each compartment. By isolating and separating peptides, this process allows for the targeted application of peptide fractions depending on the specific properties required, such as antimicrobial activity, maximizing the overall effectiveness of bioactive peptide recovery [2].

### 3.3. Antimicrobial Activities

#### 3.3.1. Antibacterial Activity of the Recovered Solutions

The peptides recovered from the tested compartments did not exhibit any antibacterial activity even after separation and purification with electrodialysis, indicating a lack of inherent antibacterial properties of the peptides present in the fractions against the examined strains as per represented in Table 4. This observation aligns with earlier observations and previous findings of Damen et al. (2024) and Huang et al. (2020) [5,38]. While some studies have shown other known antibacterial peptides, produced in different enzymatic conditions and derived from kappa-casein, beta-casein, alpha-lactalbumin, lactoferrin, and alpha S1-casein active against certain strains like *E. coli*, *B. subtilis*, *L. monocytogenes*, etc. [39,40], this absence of activity could mainly be attributed to the peptides’ ineffective interaction with bacterial membranes or other crucial bacterial components, as well as the potential presence of bacterial resistance mechanisms [41,42] and not due to their insufficient concentration as hypothesized by Damen et al. [5]. For instance, Nigenda et al. [42] demonstrated that certain cationic amphiphilic peptides, including residues such as lysine (Lys), arginine (Arg), and hydrophobic amino acids like leucine (Leu) or phenylalanine (Phe), exhibited minimal antibacterial activity against *E. coli*, which was correlated with their lower cellular toxicity compared to more active peptides. This suggests that not all cationic peptides possess the same level of antimicrobial efficacy, potentially due to differences in their amino acid composition or conformation. In the context of the current study, if the peptides recovered from the CRC compartment had insufficient affinity for the bacterial membranes due to their size, structure or charge characteristics, this could lead to ineffective membrane disruption as per demonstrated by Nigenda et al. [2]. For example, in this study, while peptides such as GPVRGPFP and QRF exhibited positive net charges, their charge density or structural conformation might have limited their ability to interact effectively with bacterial membranes through a combination of electrostatic and hydrophobic interactions, leading to membrane permeabilization [41]. Additionally, the GRAVY scores indicated varying levels of hydrophobicity, a key factor in the ability of antimicrobial peptides to insert into and disrupt bacterial lipid bilayers. Peptides with lower hydrophobicity, such as PQLE in the CRC and ELEEL in the ARC, may have been less capable of integrating into bacterial membranes, resulting in the absence of antibacterial activity. The relatively small size of these peptides (5–6 amino acids) might limit their ability to disrupt or penetrate the thicker bacterial membranes, which typically require a significant level of peptide organization or interaction to form channels or perturb the structure effectively [40]. Additionally, bacterial resistance mechanisms, such as alterations in membrane composition, may have contributed to the inactivity of the tested peptides, though this alone may not fully explain the insensitivity of all bacterial strains tested [43]. The ability of bacteria such as P. aeruginosa to degrade these peptides as a source of amino acids through aminopeptidase activity could further hinder their antibacterial efficacy [44,45]. Furthermore, the species and even the specific strains used in this study (different from those commonly reported in the literature, such as *E. coli*, *B. subtilis*, and *L. monocytogenes*) may have played a significant role in the observed insensitivity. Testing only two bacterial species limits the scope of the analysis and raises the possibility that other untested species could have been more susceptible. The presence of neutral or weakly charged peptides, such as TPV and LPLP, could have also reduced the likelihood of strong electrostatic interactions and hindered their ability to overcome bacterial defenses due to insufficient hydrophobicity, conformational limitations, or the tendency of neutral peptides to aggregate [1]. Overall, the investigation of the antibacterial properties of the peptides recovered from the tested compartments highlights the intricate interplay between peptides and bacterial cells. The absence of inherent antibacterial activity in these peptides emphasizes the need for further research to unravel the underlying mechanisms and potentially enhance the peptides’ efficacy against tested and new bacterial strains.

#### 3.3.2. Antifungal Activity

**Figure 7:**
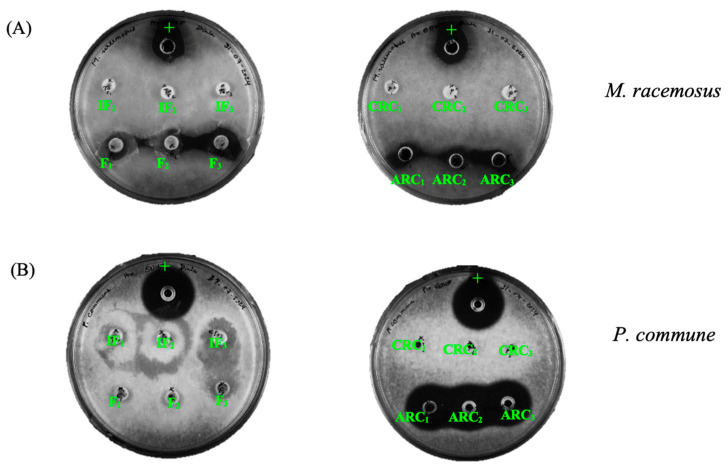
Agar well diffusion test results against Mucor racemosus (**A**) and Penicillium commune (**B**), with natamycin as the positive control (+) for initial hydrolysate (IF) and recovered feed (F), CRC, ARC after 180 min of EDUF process.

Antifungal activity was observed only after the EDUF process. Results showed inhibition against both mold strains in the ARC fraction and recovered feed fraction against *M. racemosus*, while no activity was detected in the other fractions tested (Figure 7). Significant differences in antifungal activity among the different fractions were first observed using the agar diffusion method. *M. racemosus* was significantly less sensitive (*p* < 0.05) to the ARC fraction (8 ± 1 mm) compared to *P. commune* (15 ± 1 mm) for the same fraction, representing an 87.5% larger inhibition zone (Figure 7). The recovered feed fraction exhibited an average inhibition zone of 8 ± 1 mm against M. racemosus, whereas this fraction showed no inhibitory effect against *P. commune*. The minimum inhibitory concentration (MIC) test supported these results, revealing a MIC of 0.156 mg/mL for the active fractions against both strains and 2.5 mg/mL for the initial hydrolysate showed. The MIC for the active fractions after EDUF was 16 times more potent than MIC observed in the initial hydrolysate and feed solution confirming an earlier study results where the hydrolysate was tested after 240 min of hydrolysis with Pronase E [5]. Furthermore, the pH of the fractions was measured and found to be 7.5, ruling out the possibility that inhibition was caused by extreme acidity or alkalinity; the observed antifungal effects are thus attributed to the peptides and not to pH or other external factors. These results also highlight the effectiveness of the electrodialysis process in concentrating peptides with antifungal activities.

Moreover, the distinct responses of *M. racemosus* LMA-722 and *P. commune* LMA-72 can be explained by their unique structural characteristics [5]. The EDUF process successfully concentrated antifungal peptides in the anionic solution, which interacted effectively with *M. racemosus*. However, the electrodialysis process managed to isolate specific anionic peptides that could inhibit *P. commune*, showing activity only in the anionic solution. This underscores the significance of EDUF in selectively concentrating peptides with antifungal activities. The similarity in their peptide populations may account for their comparable efficacy against fungal strains. These activities of the peptides in the ARC and feed compartments was likely enhanced by specific structural and conformational features, particularly their amphipathic nature. Amphipathic peptides, such as ELEEL and DEL, possess both hydrophobic and hydrophilic regions, which enabled them to interact more effectively with fungal cell membranes. The hydrophobic regions of these peptides likely inserted into the fungal lipid bilayer, while the hydrophilic regions interacted with the aqueous environment, leading to membrane destabilization and disruption [45]. Additionally, the relatively short sequences and flexible structures of these peptides may have facilitated their ability to penetrate and disrupt fungal membranes more efficiently through different mechanisms such as the carpet mechanism which involves monomers or oligomers of peptides accumulating parallel to the membrane surface or coarse-grain molecular dynamics which relies on membrane permeabilization by low molecular weight peptides, without causing its destruction [46]. Peptides like MKEGIHAQQ, despite its size and its inability to form an alpha helix, may have adopted a conformation that allowed partial interaction with ergosterol or other fungal-specific membrane components due structural flexibility, contributing to its antifungal activity [45]. Some anionic antimicrobial peptides are known for their antifungal activity by targeting anionic components in fungal cell membranes, leading to membrane disruption and cell lysis [45,47]. Unlike previous studies focusing primarily on antibacterial effects or enzymatic conditions alone [5,42], the present work demonstrates enhanced antifungal activity specifically after EDUF separation, in line with the hypothesis by Kadel et al. (2021) that membrane-driven separation can improve bioactivity by concentrating functional peptides [13]. The interaction of these anionic peptides with the anionic components of fungal membranes including phospholipids and ergosterol, was crucial for their antifungal efficacy. Overall, the antifungal activities of the ARC and feed peptides against the tested mold strains suggest their potential as antifungal agents. In addition, although direct experimental evaluation of the antifungal mechanism was not conducted in this study, potential mechanisms were inferred based on known interactions of antimicrobial peptides with fungal membranes. For instance, the amphipathic nature, net charge, and GRAVY scores of peptides such as ELEEL and DEL suggest their ability to interact with fungal lipid bilayers via the carpet model or toroidal pore formation, consistent with known antifungal peptide action [45,46]. The antifungal activity observed in ARC and feed fractions may be attributed to the amphipathic properties of certain peptides, which can interact with fungal membranes via mechanisms such as the carpet model or toroidal pore formation. These peptides can destabilize the membrane by aligning along its surface or forming pores that lead to leakage and cell death. Others may interact with ergosterol or phospholipid components unique to fungal membranes. Such mechanisms have been reported for short anionic and neutral antifungal peptides in the literature [45,47], suggesting that EDUF separation not only concentrates peptides but also selects for structurally favorable antifungal candidates. Future studies will be required to validate these hypotheses through mechanistic assays, including membrane permeabilization tests or ergosterol-binding interactions, as well as screening across a broader range of fungal species to confirm their efficacy and general applicability

## 4. Conclusions

This study demonstrated for the first time that white wastewater (WW), much like whey protein concentrate (WPC), was a suitable source of antifungal peptides that could be released through hydrolysis with Pronase E, and their concentration/bioactivity increased after separation by the EDUF process. The WW hydrolysate was successfully fractionated into two distinct compartments, CRC and ARC, while the migration of peptides through the EDUF process was efficient. After each compartment was analyzed, distinct peptide populations were observed that migrated based on their charge and size: 20 peptides in CRC and 9 peptides in ARC. This peptide identification through UPLC-MS/MS analysis confirmed these differences. On the other hand, while peptides recovered from CRC and ARC compartments did not exhibit antibacterial activity against Clostridium tyrobutyricum and Pseudomonas aeruginosa strains, peptides from ARC and final feed compartments displayed antifungal activity against *Mucor racemosus* LMA-722, with ARC peptides also showing activity against *Penicillium commune* LMA-72. To the best of our knowledge, this is the first study to report that the EDUF process enhances the antifungal activity of a peptide fraction derived from WW milk proteins.

Further research is required to gain deeper insights into the mechanisms underlying peptide activity and to enhance the recovery of functional peptides from WW. For that, works are currently underway on (1) optimizing the EDUF fractionation process with a membrane configuration tailored to improve the recovery of anionic peptides, potentially increasing the yield of the antifungal fraction, and (2) synthesizing and testing individual peptides to evaluate their biological activities, elucidate their mechanisms of action through membrane interaction assays, and conduct molecular docking studies to investigate their potential interactions with fungal membrane components or receptor targets. Consequently, integrating these peptides into a circular economy framework could facilitate resource recovery by transforming wastewater into a valuable source of bioactive compounds.

## Figures and Tables

**Table 1:** Summary of the statistical analyses performed throughout the study, including the tests used, the values of F or t, degree of freedom (df), and corresponding *p*-values.

Parameter	Statistical Test	F or t	df	*p*-Value
Peptide concentration (CRC)	Linear regression	F = 95.62	1, 13	<0.0001
Peptide concentration (ARC)	Linear regression	F = 94.01	1, 13	<0.0001
Peptide yield (CRC)	Cubic regression	F = 52.69	3, 1	0.1009
Peptide yield (ARC)	Cubic regression	F = 37.22	3, 1	0.1198
Global resistance over time	Quadratic regression	F = 2236.69	2, 16	<0.0001
Peptide migration rate	Quadratic regression	F = 54.45	2, 2	0.0180
Protein concentration (CRC)	Quadratic regression	F = 47.18	2, 2	0.0208
Protein concentration (ARC)	Quadratic regression	F = 50.39	2, 2	0.0195
Membrane conductivity (AEM)	Paired *t*-test	t = 5.23	2	<0.05
Membrane conductivity (CEM)	Paired *t*-test	t = 4.87	2	<0.05
UFM thickness (UFM1 vs. UFM2)	One-way ANOVA	F = 1.22	2, 6	>0.05

**Table 2:** Membrane thickness and electrical conductivity values before and after EDUF.

	Thickness (mm)		Conductivity (mS/cm)	
Membrane	Before	After	Before	After
*AEM*	0.138 ± 0.001 a*	0.140 ± 0.002 a	5.91 ± 0.12 a	4.83 ± 0.12 b
*CEM*	0.143 ± 0.002 a	0.136 ± 0.004 a	8.65 ± 0.27 a	7.09 ± 0.32 b
*UFM 1*	0.184 ± 0.001 aA	0.184 ± 0.001 aA	9.74 ± 0.31 aA	9.49 ± 0.26 aA
*UFM 2*	0.191 ± 0.002 aA	0.185 ± 0.005 aA	10.01 ± 0.27 aA	9.52 ± 0.22 aA

* Values are presented as the mean ± standard deviation for thickness and conductivity values measured before and after the sEDUF process. Lowercase letters indicate significant difference based on paired *t*-tests (*p* > 0.05) before and after the EDUF treatment. Uppercase letters indicate significant difference in conductivity and thickness between UFM 1 and UFM 2 before and after EDUF treatment based on one-way ANOVA followed by a Tukey HSD test (*p* > 0.05) (SigmaPlot 15.0 by Systat Software).

**Table 3:** Characterization of peptides recovered in the cationic (CRC) and anionic (ARC) recovery compartments following electrodialysis with ultrafiltration (EDUF) process: corresponding UV peak, analysis of molecular mass, net charge at pH 7, GRAVY score, and isoelectric point (pI).

Peak #	Sequence Name	Molecular Mass (Da)	Source	Isoelectric Point	Gravy Score	Net Charge at pH 7
	**CRC**					
**1**	SRYP	521.26	α Lactalbumin	8.46	1.00	0.99
**1**	EALG	388.20	α Lactalbumin	4.05	−1.10	−1.01
**1**	QKP	371.22	κ casein (45–47)	8.75	−3.00	0.99
**1**	VYP	377.19	α Lactalbumin	5.97	−1.27	0.00
**1**	KPAA	385.23	κ casein (63–66)	8.75	−0.48	0.99
**5**	TPV	319.16	Bsa (472–474)	5.18	0.63	0.00
**5**	MKEGIHAQQ	1040.51	Casein α s1 (123–131)	6.50	−1.09	0.09
**5**	QRF	449.24	α Lactalbumin	11.06	0.23	1.00
**5**	VDPVN	542.34	α Lactalbumin	3.09	0.04	−1.00
**5**	AHK	354.19	α Lactalbumin	10.13	0.67	1.09
**5**	KPDP	455.23	BSA (122–125)	5.84	−2.65	−0.01
**7**	YPEL	520.25	Casein α s1 (146–149)	4.05	−0.65	−1.01
**7**	VPQK	470.29	Casein β (173–136)	8.72	−1.20	1.00
**7**	YVE	409.27	α Lactalbumin	3.27	−0.27	−1.00
**8**	PQLE	485.25	Casein α (107–110)	4.60	−1.20	−1.00
**8**	KIPA	427.28	Β lactoglobulin (77–80)	8.75	0.20	0.99
**8**	QPEV	471.23	α Lactalbumin	3.27	0.43	−1.00
**12**	GPVRGPFP	825.45	Casein β (199–206)	9.75	−0.39	1.00
**12**	LPVP	424.22	Casein β (171–174)	5.53	1.20	0.00
**12**	LPLP	434.21	Casein β (135–138)	5.53	1.10	0.00
	**ARC**					
**1**	VPQ	342.18	BSA (420–422)	5.49	−0.30	0.00
**2**	KVPQ	476.21	BSA (419–422)	8.75	−1.20	0.99
**4**	DEL	375.17	Casein β (43–45)	4.05	−1.07	−2.00
**4**	DEALEK	703.34	Β lactoglobulin (130–135)	4.14	−1.47	−2.00
**5**	MKEGIHAQQ	1040.51	Casein α s1 (123–131)	6.50	−1.09	0.09
**6**	PQYLKT	748.36	α Lactalbumin	9.67	−0.22	1.00
**9**	GDLEI	545.27	Β lactoglobulin (52–56)	4.05	0.18	−2.00
**10**	ELEEL	631.31	Casein β (2–6)	4.05	−0.58	−3.00
**11**	EMPF	522.22	α Lactalbumin	4.05	−0.10	−1.00

**Table 4:** Antimicrobial activity of initial hydrolysate and different recovered solutions, based on diffusion test on agar, after EDUF process treatment against bacteria and molds.

Strains		Strain No.	Initial Hydrolysate	Recovered Feed	Cationic Solution	Anionic Solution
**Bacteria**	*C. tyrobutyricum*	ATCC25755	−	−	−	−
	*P. aeruginosa*	ATCC15442	−	−	−	−
**Molds**	*M. racemosus*	LMA722	−	+	−	+
	*P. commune*	LMA72	−	−	−	+

−: no activity; +: activity.

## Data Availability

Data will be made available on request to the corresponding author.
